# Deep Learning-Based Data Augmentation and Model Fusion for Automatic Arrhythmia Identification and Classification Algorithms

**DOI:** 10.1155/2022/1577778

**Published:** 2022-08-11

**Authors:** Shuai Ma, Jianfeng Cui, Weidong Xiao, Lijuan Liu

**Affiliations:** ^1^Xiamen University of Technology, School of Computer and Information Engineering, Xiamen 361024, China; ^2^Xiamen University of Technology, School of Software Engineering, Xiamen 361024, China

## Abstract

Automated ECG-based arrhythmia detection is critical for early cardiac disease prevention and diagnosis. Recently, deep learning algorithms have been widely applied for arrhythmia detection with great success. However, the lack of labeled ECG data and low classification accuracy can have a significant impact on the overall effectiveness of a classification algorithm. In order to better apply deep learning methods to arrhythmia classification, in this study, feature extraction and classification strategy based on generative adversarial network data augmentation and model fusion are proposed to address these problems. First, the arrhythmia sparse data is augmented by generative adversarial networks. Then, aiming at the identification of different types of arrhythmias in long-term ECG, a spatial information fusion model based on ResNet and a temporal information fusion model based on BiLSTM are proposed. The model effectively fuses the location information of the nearest neighbors through the local feature extraction part of the generated ECG feature map and obtains the correlation of the global features by autonomous learning in multiple spaces through the BiLSTM network in the part of the global feature extraction. In addition, an attention mechanism is introduced to enhance the features of arrhythmia-type signal segments, and this mechanism can effectively focus on the extraction of key information to form a feature vector for final classification. Finally, it is validated by the enhanced MIT-BIH arrhythmia database. The experimental results demonstrate that the proposed classification technique enhances arrhythmia diagnostic accuracy by 99.4%, and the algorithm has high recognition performance and clinical value.

## 1. Introduction

Arrhythmia is the main cause of various heart diseases and poses a great threat to human health. According to the WHO's 2020 report, heart disease has become the most common cause of death [1]. Among them, heart disease caused by arrhythmia accounts for 80% [2]. The electrocardiogram (ECG) is used to classify arrhythmias, which is the basic basis for cardiac disease diagnosis [3]. In routine medical diagnosis, it is very necessary to explore and study the important information in ECG, but to accurately classify ECG data for arrhythmia requires cardiologists to have rich clinical experience and professional knowledge, which will cost a lot of time and effort. As a result, utilizing a computer as an auxiliary tool to automatically detect, identify, and classify arrhythmias can provide objective diagnostic results while also saving the doctors' time [4].

Computer-aided diagnosis has been increasingly popular in the field of arrhythmia in recent years, as artificial intelligence technology has matured [5, 6]. In computer-aided diagnostics, machine learning and deep learning techniques are frequently utilized. The machine learning method first preprocesses the ECG data and then extracts ECG features through linear discriminant analysis (LDA), principal component analysis (PCA), wavelet transform (WT), independent component analysis (ICA), and other methods. Finally, the extracted ECG features are input into the classifier to complete the classification [7–13]. Classifiers include support vector machines (SVMs), decision trees, and artificial neural networks [14–16]. Machine learning methods have the advantage of being interpretable, but the models are less capable of self-learning and often fail to learn underlying the abstract patterns. At the same time, the feature extraction of machine learning requires sufficient manual intervention, and the process of extracting and selecting features takes a long time. Due to the ECG signal being affected by noise and human body variances, the generalization ability of the machine learning method is weak, and the ideal classification effect cannot be achieved.

To address the drawbacks of machine learning methods, models of deep learning are widely used for medical image recognition, where convolutional neural networks and long-short memory networks are widely used for medical image recognition, such as cancer diagnosis [17] and organ localization [18]. The application of CNN, LSTM, and GAN networks in the classification of arrhythmias can help doctors make accurate diagnoses more easily. The adequacy of data determines the performance of convolutional neural network models [19]. In general, more data indicates better recognition performance of the model [20]. On the problem of MIT-BIH data imbalance, scholars at home and abroad have proposed many methods to solve the data imbalance problem, such as through resampling and modifications at the algorithm level. Among them, resampling includes upsampling and downsampling. Upsampling is to enlarge the ECG image and insert new elements between the pixel points based on the original ECG image pixels using a suitable interpolation algorithm. Although good results can be produced by using upsampling, the upsampling method can lead to overlearning of the classifier, while the downsampling method can generate thumbnails of ECG images, which can lead to the loss of ECG data information at that time. Improved at the algorithm level, by adjusting the ratio of samples, the basic idea is similar to resampling, which also fails to fundamentally solve the problem of missing data in ECG signals. As an effective data augmentation method, GAN has been frequently utilized to address the issue of data distribution imbalance. Goodfellow et al. [21] first proposed a generative adversarial network model in 2014. Two neural networks, a generator, and a discriminator compete in generative adversarial networks to create data with a similar distribution to the original data. Afterward, GANs have been widely used for database augmentation [22–24]. GAN has been widely used as an effective data improvement method to overcome the problem of imbalanced data distribution [25]. To address the issue of data scarcity, this paper uses GAN to augment the ECG signals of scarce arrhythmia types. The generated ECG signal has morphological properties similar to the original ECG signal. To understand patient-specific ECG signals, we designed a generative adversarial network ECG-GAN that does not require any subject-specific ECG labels to train to generate arrhythmia-specific ECG signals.

This paper makes three major contributions: (1) To overcome the problem of data imbalance in the MIT-BIH arrhythmia database, we use an ECG-GAN-based data augmentation method to expand the database. By using the ECG-GAN model to expand the data on 4 types of arrhythmias, sufficient data support was provided for the classification model. (2) Because of the periodicity of ECG signals, we propose a ResNet-based spatial information fusion model and a BiLSTM-based temporal information fusion model. The model employs the ECG feature map created by the local feature extraction phase, and the model's BiLSTM network in the global feature extraction part successfully integrates neighbors' position information and achieves global feature correlation through multi-space autonomous learning. Effectively improve the performance of model arrhythmia classification. (3) The model introduces an attention mechanism to enhance features for arrhythmia-type signal segments. This mechanism can effectively focus on the extraction of key information, and form feature vectors for final classification. According to the findings, the model can greatly increase automated arrhythmia classification accuracy.

## 2. Related Work

### 2.1. Generating Synthetic ECG Signal

Due to the sensitivity of medical data, creating a large-scale ECG database utilizing medical data is extremely challenging. The problem of sample imbalance is very common in medical image diagnosis tasks [26]. The imbalanced ECG database consists of a majority type with larger data than other types and a minority type with smaller data than other types. If the machine learning model is trained with an unbalanced ECG database, the model will be biased towards the majority type, and the recognition performance of the minority type will be lower than the majority type. In recent years, the problem of unbalanced ECG data is mainly solved by synthesizing the ECG data. Traditional approaches and deep learning techniques are mostly used to synthesize ECG data. The traditional method is to synthesize ECG data by manually extracting ECG signal features and building a generative model. For example, the earliest synthetic ECG technique was proposed by McSHarry et al. [27] in 2003, who generated ECG waveforms based on calculus equations and Gaussian models. Roonizi et al. [28] introduced a polynomial spline modeling model to generate ECG data. The above traditional methods require manual extraction of features and tuning of model parameters. Personal experience will affect the quality of generative models, and subjective awareness will lead to unobjective generated results.

A deep neural network is used to generate ECG data using deep learning methods. The generative adversarial network model is a strong deep learning-based generative model that has demonstrated superior performance in a variety of domains, including picture production, resolution enhancement, and natural language processing. Golany et al. [29] improved classification performance by adding synthetic ECG heartbeats produced by standard GANs to the training set. Hernandez-Matamoros et al. [30] employed a Bi-RNN model to synthesize numerous beat signals that were identical to the original data; however, the ECG signal was not subjected to stringent ECG signal denoising, QRS wave identification, or heartbeat segmentation in the data preparation step. Zhu et al. [31] proposed a BiLSTM-CNN GAN for generating ECG signal models. BiLSTM was used as the generator in the new network model and CNN as the discriminator, but the experiment only performed data enhancement for one signal. Wulan et al. [32] used the STFT-based SpectroGAN and WaveletGAN models to generate three types of ECG signals: normal heartbeat, left bundle branch block, and right bundle branch block, but the length of the training samples involved in the experiment was short, it is not conducive to generating long valid data.

### 2.2. Deep Learning Classification Models

The two most often used neural network models are CNN and LSTM. Cui et al. [33] proposed a feature extraction method that combines traditional classification methods and CNN to improve the accuracy of arrhythmia classification by finding the best feature set. Acharya et al. [34] created a 9-layer CNN model that uses an ECG segment as an input to automatically categorize arrhythmias into five types. To identify electrocardiogram (ECG) data, Yildirim et al. [35] introduced a deep bidirectional LSTM network-based wavelet sequence model. Although the model has high performance, the database used in the experiment is too small to process a large amount of data. Swapna et al. [36] model fusion of CNN and LSTM, which integrates LSTM into the CNN model, but the classification accuracy is only 83.4%. Zhou et al. [37] proposed modeling the same ECG segment using numerous separate LSTMs and CNNs, then fusing the findings of select LSTMs and CNNs using inference rules. It could only distinguish between premature ventricular contractions induced by normal heartbeats, despite its 99.4% accuracy.

Attention-based CNNs and LSTMs are commonly used in recommender systems, activity recognition, image analysis, etc [38]. Since the attention mechanism can effectively focus on the extraction of key information, more and more scholars apply it in the direction of ECG. To merge multi-view data from CT scans for discriminative feature extraction, Zhang et al. [39] built a multi-view weighted fusion attention. Hammad et al. [40] designed a ResNet-Attention residual convolutional neural network for human identity verification. Zhang et al. [41] constructed multiple CNN-BiLSTM networks with an attention mechanism for mental stress detection by introducing an attention mechanism to the CNN and BiLSTM layers, respectively, and simultaneously adding an attention mechanism to the CNN and BiLSTM layers. Zhang et al. [42] introduced the STA-CRNN neural network model, which combines a spatiotemporal attention mechanism with a convolutional recurrent neural network to categorize nine different types of arrhythmias.

In summary, the findings of the preceding study are instructive; however, they fall short of achieving high classification accuracy and resolving the data imbalance problem. As a result, the GAN network is used in this article to solve the problem of unbalanced ECG data, as well as incorporate an attention mechanism into the ResNet-BiLSTM model to improve arrhythmia detection accuracy and optimize the classifier.

## 3. Materials and Methods

### 3.1. Arrhythmia Database

This paper uses the open-source arrhythmia database for research. There are currently four open databases for ECG signal research in the world, namely, MIT-BIH database [43], AHA database [44], CSE database [45], and ST -T database [46]. Because the MIT-BIH database is regularly utilized for arrhythmia research, it was chosen for this study. The MIT-BIH database not only contains heartbeat annotations from medical experts but also records almost all common types of arrhythmias. The database contains 48 ECG records from 47 individuals. The 48 ECG signal recordings can be classified into two groups. The first category is a total of 23 records numbered 100 to 124, which are common ECG patterns. The second category is a total of 25 records numbered 200 to 234, including clinically uncommon types of arrhythmias, but the ECG data of abnormal beats only account for one-third of all ECG data.

To unify the feature extraction and classification standards of different patients, this paper uses the AAMI standard to classify arrhythmias into 5 types and takes 44 records from the database, of which 22 are training sets and 22 are test sets.  Table 1 lists the different types of heartbeats recorded in the MIT-BIH arrhythmia database, according to the AAMI standard. In the MIT-BIH database, the number of normal heartbeats much outnumbers the other types. There are more than 90,000 different forms of normal heartbeats, but only about 800 different types of Q-type heartbeats. Arrhythmia data is much smaller than normal ECG data, and the entire database is unbalanced. Such highly imbalanced databases tend to result in very low sampling rates for minority classes [47]. To solve the issue of data imbalance, we created the ECG-GAN model to supplement the arrhythmia database's limited data.

Although the MIT-BIH datasets were used in some studies, their classification results were not as high as the model shown in this paper. Because the samples in some of the databases they utilized were too tiny, the model's capacity to detect a small number of irregular heartbeats was harmed. After data balancing, we augmented the dataset with data from the MIT-BIH database, and the classification model was trained. The model significantly increased the model's ability to detect aberrant heartbeats. The AAMI criteria for categorizing arrhythmias into five types, as well as the number of counted heartbeats for each type, are shown in Table 1.

### 3.2. ECG Signal Preprocessing

**Figure 1:**
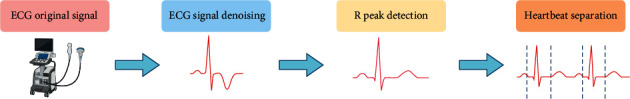
Flow chart of ECG signal preprocessing.

Noise and ECG signals are jumbled in the original data since the ECG data in the arrhythmia database is all raw data. Therefore, this paper preprocesses the ECG signal to make the signal clearer and provide a more accurate ECG signal for later experiments. Heartbeat denoising, *R*-wave detection, and heartbeat segmentation are all part of the ECG signal preprocessing. The method of ECG signal preprocessing is depicted in Figure 1.

#### 3.2.1. ECG Signal Denoising

The EGG signal has the characteristics of weak, low amplitude, low frequency, randomness, etc., and is easily disturbed by noise. However, the noise may come from the living body, such as breathing, muscle tremors, or external interference due to poor contact. Power frequency interference, electromyography interference, and baseline drift are the three primary disturbances in ECG signals.

The Discrete Wavelet Transform (DWT) is a new method for analyzing the transforms. It can be used to evaluate the signal's position in time, space, and frequency, as well as refine it over time by utilizing expansion and translation processes. Finally, the subdivision of high-frequency time and low-frequency time is realized, allowing time-frequency signal analysis to automatically adjust to the needs of the user. because the ECG signal and the noise are combined. To begin, a wavelet base function is chosen to deconstruct the noisy ECG signal, and after decomposition, the wavelet coefficients on the scale are acquired. The wavelet coefficient with a relatively big amplitude is a useful signal after the wavelet transform scale decomposes the ECG signal, while wavelet coefficients with modest amplitudes are noise. Process using threshold processing or use the threshold function to process wavelet coefficients less than the threshold. After the wavelet scale decomposition, the low-frequency coefficients and high-frequency coefficients are processed to recreate the ECG signal. Figure 1 is a flowchart of wavelet denoising. DWT is used to divide the ECG signal into high- and low-frequency sub-bands, as well as multi-level sub-bands. To produce a first-order detail coefficient, pass the ECG signal through a detail (high-frequency) filter *g*(*n*) and a down-sampler with a coefficient of 2. The coefficients of the approximation (low-frequency) and detail filters are interrelated and together they are called quadrature mirror filters. From the approximation coefficients *h*(*n*), the *g*(*n*) detail filter coefficients are calculated as follows, as shown in formula (1), formula (2), and formula (3). Because the scale function of the 6-wavelet is similar to that of the ECG signal, this paper uses db6 as the wavelet base function to perform a 5-scale wavelet transform on ECG data.

(1) $$\begin{array}{c} G\left(L-1-n\right)={\left(-1\right)}^{n}h\left(n\right), \end{array}$$

where *L* is the length of the filter's coefficients. The following is a representation of subsampling and DWT decomposition:

(2) $$\begin{array}{c} Y_{\text{low}}\left[n\right]=\sum_{k=-\infty }^{\infty }x\left[k\right]h\left[2n-k\right], \end{array}$$

(3) $$\begin{array}{c} Y_{\text{high}}\left[n\right]=\sum_{k=-\infty }^{\infty }x\left[k\right]h\left[2n-k\right]. \end{array}$$

**Figure 2:**
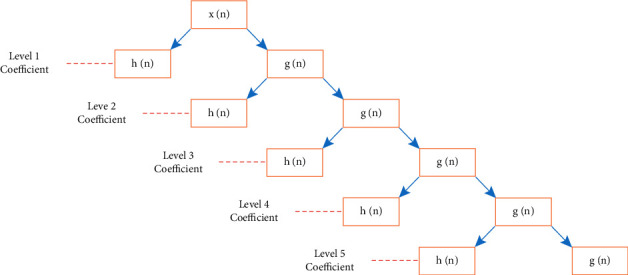
Discrete wavelet decomposition of ECG signal.

Because the sample rate of the MIT-BIH ECG signal is 360 Hz, the maximum frequency of the original ECG signal is below 180 Hz, according to the Nyquist sampling theorem. As a result, the maximum frequency of the *D*1 layer for signal decomposition is 180 Hz. After decomposing the original signal, we can deduce that the energy of the detail components in layers 1-2 corresponds to the original signal's high-frequency interference. It shows that the 1-2 layers are the main places where high-frequency noise is concentrated. Therefore, we need to filter out the detail components of the *D*1 and *D*2 layers and achieve the purpose of removal by setting them to 0. Then, the 3∼5 layers of wavelet coefficients obtained by decomposing the signal are used to process the threshold value of the signal through the soft threshold formula. The pywt threshold () function provides threshold filtering, and the default is soft threshold filtering with mode = “soft”. To obtain the denoised signal, the wavelet coefficients are finally inversely converted. Therefore, this paper takes the db6 wavelet as the mother wavelet.  Figure 2 shows the process of decomposing an ECG signal using discrete wavelets. In Figure 2, *x* (*n*) is a discrete input signal, *g*(*n*) is a low-pass filter used to filter high-frequency information in the ECG signal and output low-frequency information, and *h*(*n*) is a high-pass filter used to filter high-frequency information in the ECG signal and output low-frequency information. It is used to output high-frequency signals while filtering low-frequency ones. The signal sampling rate used in this paper is 360 HZ, and the db6 wavelet function is used as the mother wavelet to decompose the ECG signal into five layers. The ECG signal is then recreated using the inverse wavelet transform.

#### 3.2.2. R Peak Detection and Beat Separation

**Figure 3:**
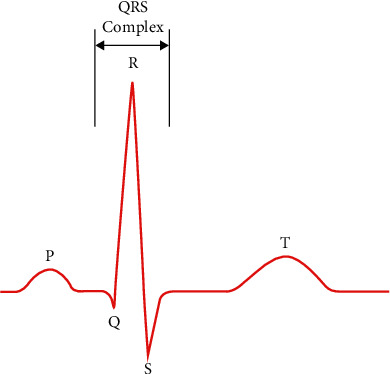
The QRS waveform.

Clinically, the heartbeat signal collected by the ECG acquisition equipment is usually several tens of seconds or longer, and a continuous signal recording usually contains many heartbeats. For some cardiac diseases, the occurrence of abnormalities may not be continuous, but in some of these heartbeats, not every heartbeat will show abnormalities. Therefore, the analysis of arrhythmias should be performed on individual heartbeats, rather than analyzing the entire heartbeat signal recording data. After the heartbeat signal has been denoised, the next step is to locate and slice the heartbeats of a continuous segment of the signal and analyze its rhythm class. The *R*-peak is the most easily identifiable waveform in a heartbeat, with the most distinctive features such as amplitude and morphology. It is feasible to utilize the position of the *R*-peak as a reference to discover additional distinctive points by acquiring information on its location.  Figure 3 depicts the QRS waveform. After processing the denoised ECG signal, the feature information of the QRS waveform group was retrieved using the Pan–Tompkins technique. A total of 150 points samples are intercepted for one heartbeat by finding the *R*-peak location and intercepting 50 points forward and 100 points backward from the *R*-peak position. A huge heartbeat cycle is included in the size.

### 3.3. ECG Data Enhancement

Goodfellow et al. [21] introduced a generative adversarial network framework in 2014, which uses adversarial neural processes to estimate generative models. The generative adversarial network is a sort of unsupervised learning with two components: generator *G* and discriminator *D*. The generator and discriminator engage in a continuous game throughout the training phase. The discriminator *D*'s purpose is to correctly discriminate the input into the discriminator, whereas the generator *G*'s goal is to generate a new image that is comparable to the real image, whether or if the image is genuine. In the optimal state, the generator *G* may generate pictures that the discriminator *D* cannot identify between real and false as the number of training rounds increases. The generative adversarial network's loss function is represented in formula (4).

(4) $$\begin{array}{c} \underset{G}{\min}\underset{D}{\max}V\left(D,G\right)=E_{x∼P_{\text{data}\left(x\right)}}\left[\log D\left(x\right)\right]+E_{x∼P_{z}\left(z\right)}\left[\log\left(1-D\left(G\left(z\right)\right)\right)\right]. \end{array}$$

This formula consists of two terms, the true image is represented by *x*, the noise input to the generator *G* is represented by *z*, and the image generated by the generator *G* is represented by *G*(*z*). As a result, the loss function's optimization goal is to reduce the loss of the generator *G* while maximizing the loss of the discriminator *D*.

**Figure 4:**
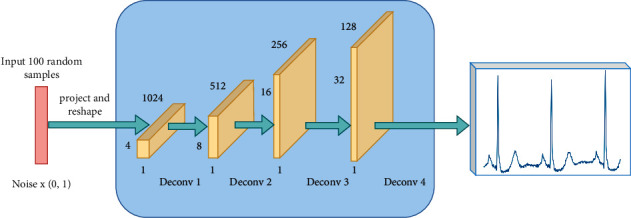
ECG-GAN generation model.

An input layer, four deconvolution layers, and an output layer make up the ECG-GAN generative model employed in this paper. Different from the two-dimensional and three-dimensional data in the model that generates pictures, the ECG signal is one-dimensional data, so the deconvolution layer in the generated model in this paper is one-dimensional, and the specific deconvolution structure is generated as shown in Figure 4. In each deconvolution operation, the features of the previous step will be enlarged by the corresponding multiples in the upsampling step. For example, when the upsampling parameter UpSampling 1D is set to 5, the feature map will be enlarged by 5 times accordingly, which can be combined into a new feature. The aim of this process is to extract more information and increase the quality of the heartbeats that are created. Except for the final output layer, the activation function of the generative model in ECG-GAN adopts the ReLu function. At the beginning of training the generative model, a random vector with a size of 100 dimensions that obeys the normal distribution is input into the generative model, and in the process of deconvolution, the 100-dimensional noise random vector is reshaped into 1*∗*128-dimensional features, the convolution kernel in each layer's deconvolution layer is 6 pixels wide, and “same padding” is used. The number of channels is lowered to half of the previous layer by layer after the deconvolution operation, but because each step is conducted upsampling, the size of the feature map grows proportionately. The synthetic heartbeat data is eventually generated in the last layer, which outputs a feature map with a channel number of 1.

**Figure 5:**
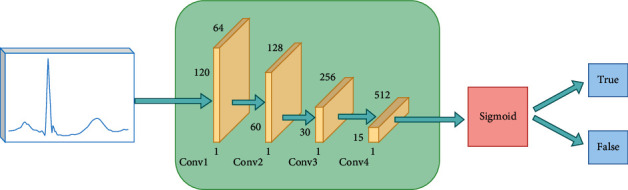
ECG-GAN discriminant model.

An input layer and four output layers comprise the ECG-GAN discriminative model. The ECG-GAN discriminative network is shown in Figure 5, with the exception that the Sigmoid function is used to activate the output layer, while the other layers use the LeakyReLU function. After inputting the real heartbeat data and the generated heartbeat data into the discriminant model, through the convolution layer in the model, to determine if the input is true or false, the classification function returns a probability value of 0∼1. The model's learning rate is 0.1, and there are 1000 iterations. To make the model optimal, this paper uses Adam to adjust the model parameters. In addition, to prevent the discriminator's discriminative ability from being too strong, the generator cannot reach a balance with it, the random deactivation technique is used in the generative model, and the random deactivation coefficient is set to 0.4.

### 3.4. ECG Classification Model

**Figure 6:**
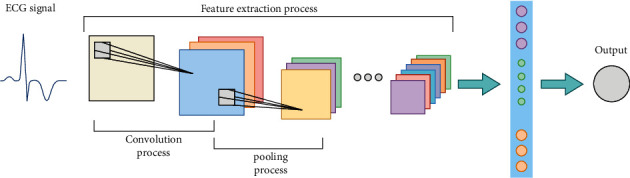
Convolutional neural network structure diagram.

**Figure 7:**
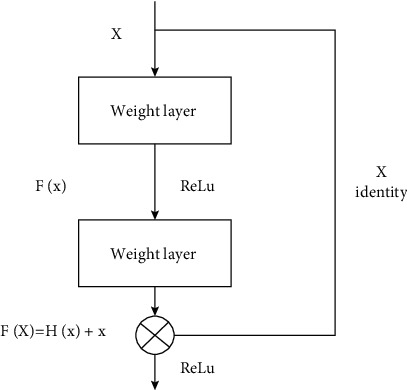
Residual block structure diagram.

Convolutional neural networks have excelled in many areas, particularly image identification [22, 23]. Convolutional neural networks [24, 25] are a sort of feedforward neural network with four layers: an input layer, a convolutional layer, a pooling layer, and a fully connected layer, and the network has features such as weight sharing and local connectivity. The network takes the preprocessed ECG data and automatically extracts the features of the ECG signal, and the process of ECG signal feature extraction is performed by sliding multiple convolutional windows over the ECG image and performing convolutional operations on the local ECG features, where the network needs to compute additional ECG feature mappings in order to be able to detect multiple local features. Therefore, a complete convolutional layer consists of several feature mappings, which can extract more ECG features and finally complete the ECG feature extraction.  Figure 6 depicts the convolutional neural network's structure. Since the gradient explosion problem and the network degradation problem are impossible to avoid as the model structure becomes complex and cumbersome, the introduction of residual blocks in the deep network structure can effectively solve the gradient disappearance and gradient explosion problems, which in turn can make the model have better performance.  Figure 7 shows the structure of the residual block.

**Figure 8:**
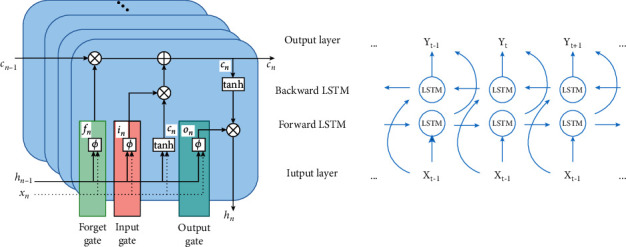
LSTM memory unit and BiLSTM structure.

The LSTM is a variety of recurrent neural networks that uses specific gate computation to learn long-term associations to solve the problem of unstable gradients in the recurrent neural networks. A set of recurrently connected memory units makes up the LSTM architecture. Although the LSTM network has the same topology as a traditional recurrent neural network, the hidden layer neurons are replaced with recurrently connected memory cells.  Figure 8 depicts the LSTM memory cells and BiLSTM structure. Each LSTM memory cell contains one or more self-connected memory cells and three multiplication cells, i.e., forget gate *f*_*n*_, input gate *i*_*n*_, and output gate *o*_*n*_, giving the cells continuous write, read, and reset operations. The forgetting gate determines the information discarded and retained from the cell state, and its purpose is to provide a way for the memory cell to reset itself, which is essential for tasks that require the network to forget previous inputs; the input gate selectively adds fresh information to the cell state and updates it, while the output gate ensures that the current neuron's output is passed on to the next neuron.

Below are the LSTM memory unit's cell and output states, as well as the calculation formula (5)–(10) for each gate :

(5) $$\begin{array}{c} \;f_{n}=\varphi \left(b_{f}+\mu_{f}^{T}x_{n}+\omega_{f}^{T}h_{n-1}\right), \end{array}$$

(6) $$\begin{array}{c} i_{n}=\varphi \left(b_{i}+\mu_{i}^{T}x_{n}+\omega_{i}^{T}h_{n-1}\right), \end{array}$$

(7) $$\begin{array}{c} \;c_{n}=f_{n}c_{n-1}+i_{n}c_{n}, \end{array}$$

(8) $$\begin{array}{c} c_{n}=\tanh\left(b_{c}+u_{c}^{T}x_{n}+w_{c}^{T}h_{n-1}\right), \end{array}$$

(9) $$\begin{array}{c} \;h_{n}=o_{n}\tanh\left(c_{n}\right), \end{array}$$

(10) $$\begin{array}{c} o_{n}=\varphi \left(b_{o}+w_{o}^{T}x_{n}+u_{o}^{T}h_{n-1}\right). \end{array}$$

The forgetting gate *f*_*n*_ and the input gate *i*_*n*_ control the LSTM memory unit; each time unit of the sequence may then delete or add information to the memory block. The input sequence at instant *n* is represented by the equation *x*_*n*_. The output vector of the LSTM at the preceding instant is *h*_*n*−1_. The input and loop weight vectors of the output and forgetting gates, respectively, are *u*_*i*_, *w*_*i*_, *u*_*f*_ and *w*_*i* _, and the bias term is *b*. The input and cyclic weight vectors of the output gate are represented by the parameters *u*_*o*_ and *W*_*o*_. The standard LSTM model has the disadvantage of being unable to correctly collect future information and can only handle positive input. The positive and negative LSTM layers in the input data may completely consider the global information of the hidden layers in a bidirectional long and short-term memory network (BiLSTM), which consists of one input layer, two hidden layers, and one output layer. Since this paper studies ECG signals, which are temporal, the BiLSTM is more suitable for global feature extraction.

The attention mechanism achieves classification accuracy by mimicking the human brain attention mechanism in the form of capturing more critical features on input information features. The attention mechanism has demonstrated strong performance in voice and natural language processing, as well as benefits in temporal information processing [27, 28]. A mapping from a Query to a set of Key-Values may be characterized as the Attention mechanism. There are three steps in the calculation of attention in this mechanism; the first one is to obtain the relevant weights by the similarity calculation between Query and Key. The similarity calculation formulas (11)–(13) are multiplication, cascade, and perception, respectively. The SoftMax function then performs the normalizing step. To produce the final attention vector output, the weights and the matching Key are weighted and summed, where *W*_*a*_, *U*_*a*_, *v*_*a*_ are the learning parameters, *Q* is the query, and *K*_*i*_ refers to the key value.

(11) $$\begin{array}{c} f\left(Q,K_{i}\right)=Q^{T}W_{a}K_{i}, \end{array}$$

(12) $$\begin{array}{c} f\left(Q,K_{i}\right)=W_{a}\left[Q:K_{i}\right], \end{array}$$

(13) $$\begin{array}{c} f\left(Q,K_{i}\right)=v_{a}^{T}\tanh\left(W_{a}Q+U_{a}K_{i}\right). \end{array}$$

**Figure 9:**
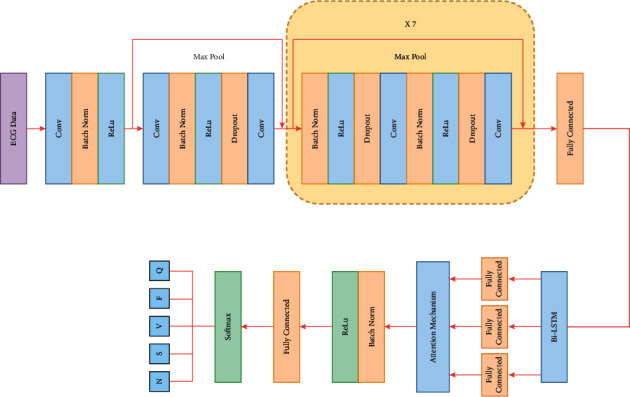
ResNet-BiLSTM-Attention model diagram.

In summary, an automatic classification model of arrhythmias based on the attention mechanism is designed in this paper using ResNet-BiLSTM.  Figure 9 depicts the graphical representation of the model. Three parts constitute the main part of the model: local feature extraction, global feature extraction, and feature reinforcement. ResNet is used to implement the local feature extraction part. The morphological elements of the original ECG signal can be successfully extracted using the convolutional operations in the convolutional neural network. When a deep neural network reaches saturation, adding more layers or neurons can lead to network degradation and poor model performance. Using residual blocks in a deep network can help solve the problem of gradient disappearance and explosion, resulting in better performance when training networks with more layers. To compress long sequences of ECG signals into shorter sequences of local feature vectors by learning local features, the model uses a stacked residual convolution module. The ECG signal is input to the present-day initial layers, and the output ECG signal features are processed sequentially by seven residual blocks, which contain 14 convolution layers and 7 MaxPool layers. Each residual block combines the output of the fast join with the output of the second convolutional layer and contains two Batch Norm, the ReLu layer, and Dropout layers. When the feature map goes across a max pooling layer with a pool size of 2, its length is cut in half. Following the local feature extraction phase, the final subsampling of the original input is carried out 28 times, and the output length is 1/256 of the input length. Then, the position information of the nearest neighbors is effectively fused using the BiLSTM model, and the retrieved local feature vectors are input to the BiLSTM one by one for global feature extraction. To extract global features, the original signal is fed into a BiLSTM algorithm, where each LSTM unit in the forward and backward layers has a number limit of 128. Global features from BiLSTM and local features from ResNet are used to become fused hybrid features, and multi-space autonomous learning is performed through an attention mechanism to obtain correlations between global and reinforcement features. Finally, the arrhythmias of *N*, *S*, *V*, *F,* and *Q* types are classified by the SoftMax layer. Meanwhile, the ResNet-BiLSTM model without adding the attention mechanism is used as a comparison experiment in this paper, so as to highlight the influence of the attention mechanism on the classification effect.

### 3.5. Evaluation Method

This paper uses three assessment measures to assess the model's classification performance: accuracy, recall, and specificity. The calculation method and significance of each index are shown in formulas (14)–(16) as follows.

Accuracy: refers to the proportion of accurately categorized true positive and true negative samples among all samples.

(14) $$\begin{array}{c} \text{Acc}=\frac{\left(\text{TP}+\text{TN}\right)}{\text{TP}+\text{TN}+\text{FP}+\text{FN}}. \end{array}$$

Sensitive: refers to the percentage of all positive samples that are positive.

(15) $$\begin{array}{c} \text{Sen}=\frac{\text{TP}}{\text{TP}+\text{FN}}. \end{array}$$

Specificity: refers to the proportion of correctly predicted abnormal heartbeats to all data that are abnormal.

(16) $$\begin{array}{c} \text{Spe}=\frac{\text{TP}}{\text{TP}+\text{FP}}.\; \end{array}$$

The number of valid classifications in the formula above is called true positives (TP). True negatives (TN) reflect the number of misclassifications, whereas false negatives (FP) and false positives (FN) measure the number of misclassifications.

## 4. Results

### 4.1. Experiment Platform

The experiments in this paper use the Python 3.8 programming language and the Pytorch deep learning framework. The operating system is 64 bit Linux, the CPU is Intel (R) Xeon (lice Lake) Platinum 8369R @ 2.90 GHz, the GPU is NVIDIA A10, and the video RAM is 24 GB.

### 4.2. ECG Signal Preprocessing

**Figure 10:**
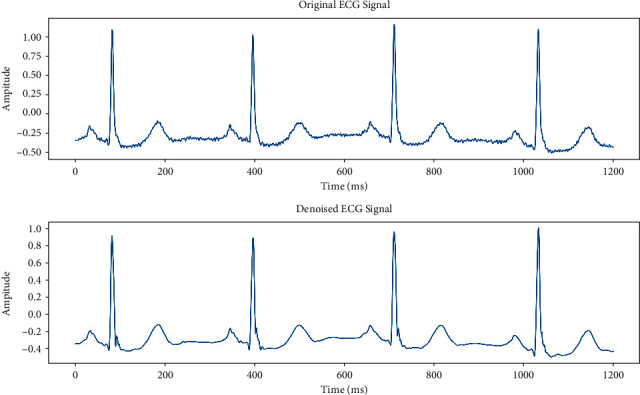
Comparison of ECG before and after denoising.

The ECG signal 101 from the MIT-BIH dataset is shown in Figure 10 as an example of denoising. The first graph shows the original ECG signal, whereas the second graph shows the denoised ECG signal. The denoised ECG signal's waveform smooths down and its quality is noticeably improved, which raises the precision of waveform recognition.

**Figure 11:**
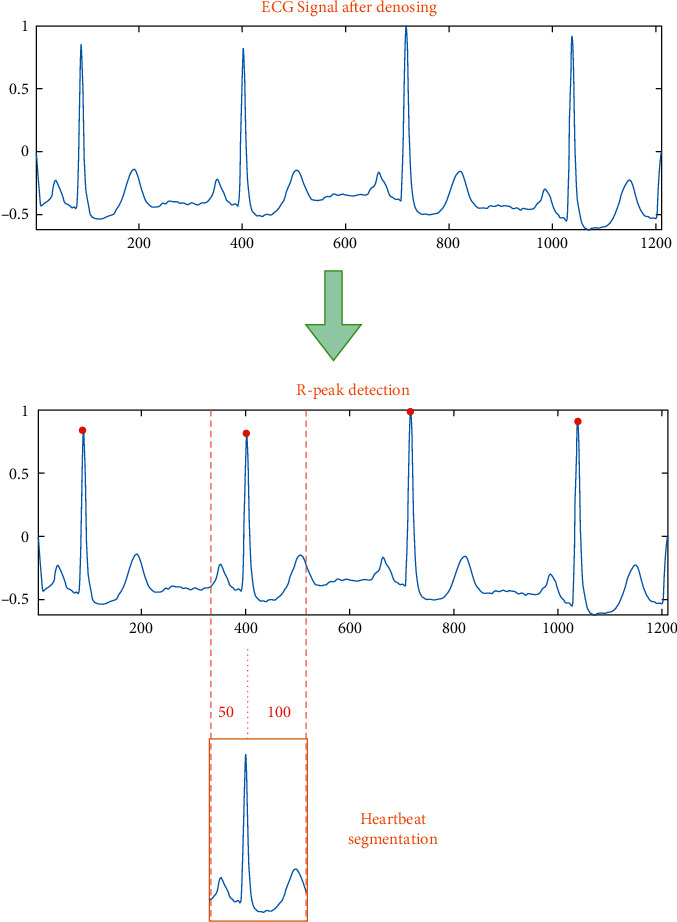
Results of QRS complex detection and heartbeat segmentation.

After the raw signal is preprocessed and the signal is denoised, the heartbeat segmentation is needed to obtain a single heartbeat signal before further analysis of the ECG signal. The most important part of heartbeat segmentation is to detect and identify the characteristic points, including the peak, start point, and end point of characteristic points. QRS is the wave with the highest amplitude and energy in the heartbeat cycle, so the most important part of heartbeat segmentation is the detection of the QRS wave. After locating the QRS wave, the R-wave peak is used as the reference point, and based on the distance to the QRS wave, the interval location of other wave peaks can be determined. To facilitate subsequent data processing and feature extraction, heartbeat segmentation we use a total of 150 data as a complete heartbeat based on the location of QRS wave peaks, taking the R-peak as the reference point, intercepting 50 sample points forward and 100 sample points backward, containing QRS peak points. The results of QRS complex detection and heartbeat segmentation are shown in Figure 11. For the purpose of this paper, we employ the MIT-BIH arrhythmia dataset, which has a 360 Hz sampling rate, a normal heart rate of 60 to 120 beats per minute, and a pulse duration of 0.5 to 1 seconds.

### 4.3. Data Enhancement and Classification

**Figure 12:**
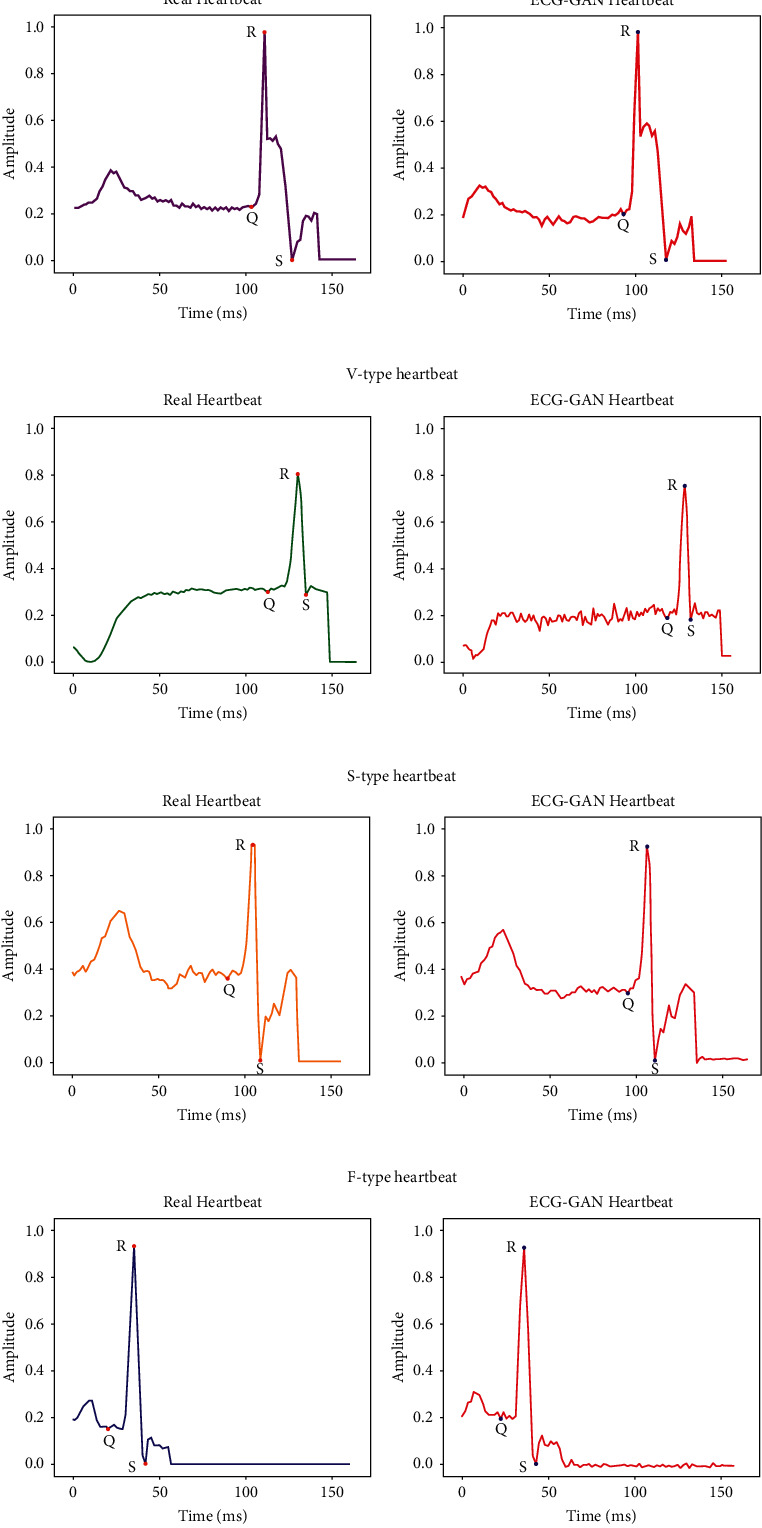
Comparison of original ECG and generated ECG.

Because the patients had pacemakers, four records from the MIT-BIH arrhythmia database were excluded from this paper. The ECG-GAN approach is used to supplement the remaining 44 pieces of data. After 10,000 training cycles with the ECG-GAN model, Figure 12 illustrates a comparison of raw and produced heartbeats. The synthesized ECG data has the same QRS waveform as the original, as shown in the figure, which can reconstruct the original ECG more realistically, thus augmenting the scarce ECG data.  Table 2 displays the differences between the original database and the expanded database. The Q-type heartbeat data is expanded to 20399, the S-type heartbeat data is expanded to 20236, the V-type heartbeat data is expanded to 20179, and the F-type heartbeat data is expanded to 20339.

**Figure 13:**
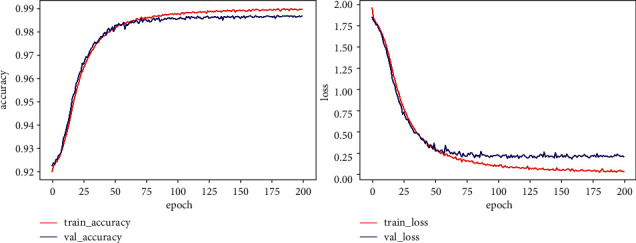
Graph depicting the ResNet-BiLSTM model accuracy and loss rates.

**Figure 14:**
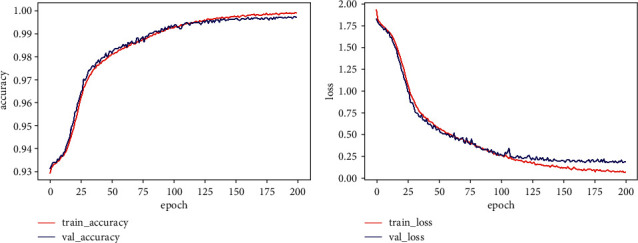
Graph depicting the ResNet-BiLSTM-attention model accuracy and loss rates.

**Figure 15:**
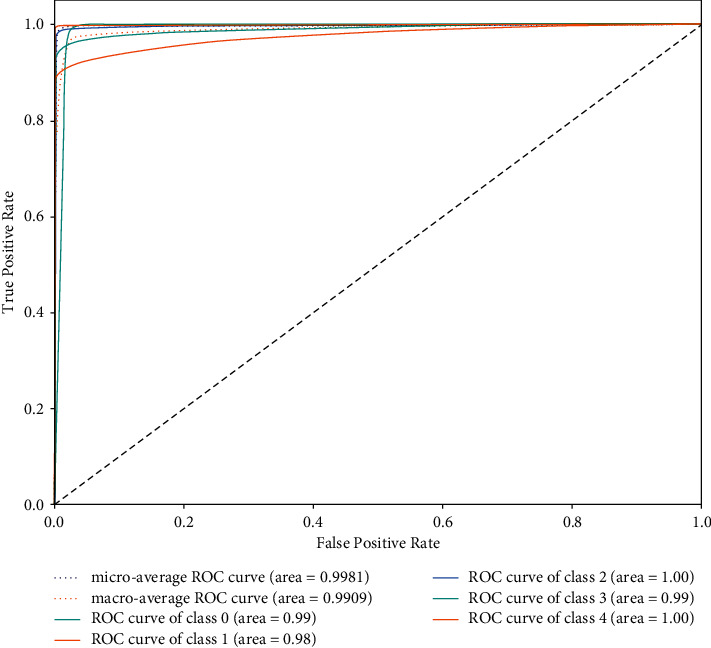
ResNet-BiLSTM-attention model ROC curve.

This paper uses ECG-GAN to generate scarce ECG data to expand the database. Under the condition of the same data, this paper sets up ResNet-BiLSTM and ResNet-BiLSTM-Attention hybrid models for comparative experiments. Tables 3 and 4, respectively, show the Acc, Spe, and Sen of the two models for five types of heartbeat types. As shown in the comparison, the ResNet-LSTM-Attention hybrid model outperforms the ResNet-BiLSTM hybrid model in terms of overall performance. In particular, the recognition of type *V* and type *F* is significantly improved.  Figure 13 shows the accuracy rate and loss rate change curves for the full ResNet-BiLSTM model process, whereas Figure 14 shows the accuracy rate and loss rate change curves for the entire ResNet-BiLSTM-Attention model process. Figures 13 and 14 demonstrate this, when the training times of the ResNet-BiLSTM model are 100, the overall accuracy curve is still in the rising stage, the loss rate curve is in the falling stage, and then gradually stabilizes; the training times of the ResNet-BiLSTM-Attention model are at 100, the overall curve of the accuracy rate and loss rate has stabilized, and it can be seen that the fitting speed of the ResNet-BiLSTM-Attention model is faster. The ResNet-BiLSTM-Attention model's ROC curve is shown in Figure 15, and it can be observed that the ROC area is almost close to 1, demonstrating the model's excellent stability and suitability for use in the creation of an automatic classification model for cardiac arrhythmias. In Figure 15, class 0 represents Heartbeat Type *N*, class 1 represents Heartbeat Type *S*, class 2 represents Heartbeat Type *V*, class 3 represents Heartbeat Type *F*, and class 4 represents Heartbeat Type *Q*.

## 5. Discussion

Due to the inherent disadvantages of ECG signals such as low frequency and susceptibility to interference, it is an extremely complex and tedious task to adopt efficient and accurate extraction of ECG features. Machine learning in the traditional sense requires the design of feature extractors to manually extract features, but due to the very limited nonlinear fitting ability of some machine learning methods, it is not always possible to extract high-level and highly differentiated ECG features very accurately. Meanwhile, the existing public ECG database has the problem of data imbalance, and some ECG data have the problem of scarcity, which will cause the omission of important ECG information when performing denoising and feature extraction, plus the different classification effects of various classifiers, thus leading to the final classification results are not good enough.

In this paper, we design an ECG-GAN network to extend the sparse data in an ECG database and a ResNet-BiLSTM-Attention classification model to type ECG data into five categories using AAMI criteria. The experimental findings reveal that all five heartbeat types have increased in accuracy and the overall results have been enhanced. ECG-GAN model data augmentation is compared with the traditional algorithm, which enlarges the ECG and inserts new elements between the original pixels on the ECG. Although this method creates thumbnails of ECG images, it results in missing ECG data. Often these missing data may be the data we need, which in turn leads to overlearning of the classifier. The ECG-GAN model designed in this paper does not need to design complex feature engineering for the generated model, but simply must create the neural network structure to detect the true characteristics of the real ECG signal which helps to generate data closer to that in terms of waveform and other feature data, and we can generate ECG features without spending much time to find the right parameters. The final classification model performs feature enhancement for arrhythmia-type signal segments by introducing an attention mechanism based on ResNet-BiLSTM. This mechanism can effectively focus on the extraction of focused information and form feature vectors for final classification. According to the outcomes of the trials, the model increases the accuracy of automated arrhythmia classification.

This paper summarizes the classification results of some previous work on arrhythmias, as shown in Table 5. Sahoo et al. [52] introduced a QRS complex feature identification technique that used the multiresolution wavelet transform (MWT) and integrated it with SVM as a classifier, achieving a 98.39% accuracy. Elhaj et al. [50] employed a combination of SVM and RBF to accurately categorize five types of arrhythmias: N, S, V, F, and Li et al. [48] designed a methodology for classifying ECG signals based on WPE and RF that has a 94.61% accuracy. All of the approaches listed above are machine learning methods that involve manual feature extraction, which is a time-consuming and complicated operation, and then use a classifier alone to complete the classification process. Acharya et al. [34] introduced a CNN-based technique that removes the need for human heartbeat signal feature extraction, simplifying the process and yielding a 94.03% accuracy. Tan et al. [49] employed CAD synthetic ECG to increase the database and categorize it with a 95.8% classification accuracy using the CNN-LSTM model. In summary, to compensate for the lack of data samples, an ECG-GAN model was established, and a ResNet-BiLSTM-Attention model was developed in this study to overcome the issue of data imbalance in the arrhythmia database. The model's accuracy was tested using the enhanced dataset, and the results showed a classification accuracy of 99.4%. The results of the literature review are summarized in the table below. In this paper, we found that type V arrhythmias include atrial premature heartbeats and junctional escape. It is easy to be misclassified into other categories and therefore has the lowest accuracy. In the next research work, more effective feature information is extracted to distinguish V-type arrhythmias and improve their classification accuracy. The heartbeat data generated by the ECG-GAN model suggested in this paper has the same form as the original data; however, the smoothness needs to be enhanced. This is related to the ECG-GAN model's instability. As a result, in the following study, we will look into how to make the training process of generative adversarial networks more stable.

## 6. Conclusions

The ECG-GAN data augmentation model and the ResNet-BiLSTM-Attention classification model are proposed in this paper. We present the ECG-GAN data improvement model for the problem of data imbalance in MIT-BIH arrhythmia data, which can efficiently tackle the problem of data imbalance. Meanwhile, for the existence of periodicity of ECG signals, we proposed ResNet-based spatial information fusion and BiLSTM-based temporal information fusion models. The model effectively fuses the location information of the nearest neighbors through the ECG feature map generated by the local feature extraction part and obtains the correlation of the global features through the BiLSTM network in the global feature extraction part, and through the multi-space autonomous learning. The model also introduces an attention mechanism for feature enhancement of arrhythmia-type signal segments. This mechanism can effectively focus on the extraction of focused information and form feature vectors for final classification. Finally, the accuracy of the classification model is tested using the MIT-BIH arrhythmia database, which has a 99.4% accuracy rate. The results of the experiments show that our proposed strategy surpasses other models in terms of overall performance, proving its superiority. The algorithm can accurately identify the type of arrhythmia with a high accuracy rate. These experimental outcomes demonstrate that the proposed technique outperforms the most recent methods, which proposes that our system for classifying arrhythmias has considerable therapeutic potential. Additionally, additional situations, such as the detection and classification of atrial fibrillation, can be handled using the proposed way in our system for classifying arrhythmias. In this paper, generative experiments on real data with other sampling frequencies were not conducted in this paper due to time constraints. Therefore, the effect of the sampling frequency of ECG data on the authenticity of ECG data generated by generative adversarial networks becomes the focus of the next work. Meanwhile, we only utilized the MIT-BIH arrhythmia database, but we hope to extend the developed model to other arrhythmia databases in the future to improve its performance.

## Figures and Tables

**Table 1:** AAMI standard classification.

Category	Annotations	Name	Fragment number
Normal (N)	Normal beat	NOR-N	90589
	Left bundle block beat	LBBB-L	
	Right bundle block beat	RBBB-R	
	Nodal (junctional)escape beat	NE-j	
	Atrial escape beats	AE-e	

Supraventricular (S)	Atrial premature beat	AP-A	2779
	Aberrated atrial premature beat	aAP-a	
	Nodal(junctional) premature beat	NP-J	
	Supraventricular premature beat	SP-S	

Ventricular (V)	Premature ventricular contraction	PVC-V	7236
	Ventricular escape beat	VE-E	

Fusion (F)	Fusion of ventricular and normal beat	Fvn-F	803

Unknown (Q)	Paced beat	P-/	8039
	Fusion of paced and normal beat	Fpn-f	
	Unclassified beat	U-Q	

**Table 2:** Summary of the 5 types of Heartbeats.

Heartbeat type	Number of raw ECG	Expanded number of heartbeats
N	90589	90589
Q	8039	20399
V	7236	20236
S	2779	20179
F	803	20339
Total	109446	171742

**Table 3:** Experiment results of ResNet-BiLSTM classification.

Classification	Evaluation parameters	Heartbeat type				
		*N*	*S*	*V*	*F*	*Q*
ResNet-BiLSTM	Accuracy/%	99.14	99.25	97.32	97.7	99.09
	Spe/%	99.93	93.54	98.12	98.1	99.46
	Sen/%	99.13	99.14	98.51	98.51	99.83

**Table 4:** Experiment results of ResNet-BiLSTM-Attention classification.

Classification	Evaluation parameters	Heartbeat type				
		N	S	V	F	Q
ResNet-BiLSTM- attention	Accuracy/%	99.96	99.55	99.06	99.21	99.32
	Spe/%	99.97	99.09	98.16	98.87	99.42
	Sen/%	99.31	100	98.25	99.42	99.67

**Table 5:** A comparison of our method to other methods.

Authors	Method	Performance		
		Acc (%)	Sen	Spe
Acharya et al. [34]	Generation of synthetic data + 9-layer deep CNN	94.0	96.7%	91.5%
Li et al.[48]	WPE + RR + RF classifier	94.6	—	—
Tan et al. [49]	CAD generation of synthetic data + CNN-LSTM	95.8	87.9%	87.9%
Elhaj et al. [50]	SVM-RBF + NN	97.0	97.1%	96.9%
Jangra et al. [51]	DWT + SVM + CNN	97.8	—	—
Sahoo et al. [52]	MWT + SVM	98.4	96.7%	98.9%
Our method	ECG-GAN + Resnet-BiLSTM-attention	99.4	98.4%	99.3%

## Data Availability

The data used to support the findings of this study are available from the corresponding author upon request.
